# Notch Signalling Is Required for the Formation of Structurally Stable Muscle Fibres in Zebrafish

**DOI:** 10.1371/journal.pone.0068021

**Published:** 2013-06-28

**Authors:** Susana Pascoal, Joana Esteves de Lima, Jonathan D. Leslie, Simon M. Hughes, Leonor Saúde

**Affiliations:** 1 Instituto de Medicina Molecular e Instituto de Histologia e Biologia do Desenvolvimento, Faculdade de Medicina da Universidade de Lisboa, Lisboa, Portugal; 2 Instituto Gulbenkian de Ciência, Oeiras, Portugal; 3 Randall Division for Cell and Molecular Biophysics, New Hunt’s House, Guy’s Campus, King’s College London, London, United Kingdom; University of Sheffield, United Kingdom

## Abstract

**Background:**

Accurate regulation of Notch signalling is central for developmental processes in a variety of tissues, but its function in pectoral fin development in zebrafish is still unknown.

**Methodology/Principal Findings:**

Here we show that core elements necessary for a functional Notch pathway are expressed in developing pectoral fins in or near prospective muscle territories. Blocking Notch signalling at different levels of the pathway consistently leads to the formation of thin, wavy, fragmented and mechanically weak muscles fibres and loss of stress fibres in endoskeletal disc cells in pectoral fins. Although the structural muscle genes encoding Desmin and Vinculin are normally transcribed in Notch-disrupted pectoral fins, their proteins levels are severely reduced, suggesting that weak mechanical forces produced by the muscle fibres are unable to stabilize/localize these proteins. Moreover, in Notch signalling disrupted pectoral fins there is a decrease in the number of Pax7-positive cells indicative of a defect in myogenesis.

**Conclusions/Significance:**

We propose that by controlling the differentiation of myogenic progenitor cells, Notch signalling might secure the formation of structurally stable muscle fibres in the zebrafish pectoral fin.

## Introduction

The development of many organs starts with the formation of a primordium at specific embryonic locations in response to combinatorial positional signals. This is the case with the appendages. The limb/fin mesenchyme precursor cells protrude from the embryonic trunk to form a small bud covered by a layer of ectoderm and will give rise to precise arrangements of differentiated cells such as cartilage/bone and muscle [1], [2]. The genetic network that triggers and controls paired fin outgrowth seems to be similar to the developmental program of the tetrapod limb until larval stages. After that, morphological and genetic differences become obvious, underlying the diversity of appendages formed in different vertebrates.

In amniote tetrapods, paired appendage outgrowth is controlled along the proximal-distal (PD), anterior-posterior (AP) and dorsal-ventral (DV) axes by three organizing centres. The apical ectodermal ridge (AER) promotes outgrowth and skeletal patterning along the PD axis; the zone of polarizing activity (ZPA) patterns the AP axis; the non-ridge ectoderm specifies the DV axis [1].

The apical ectodermal fold (AEF) of the zebrafish pectoral fin bud, like the tetrapod AER, expresses several Fgf molecules, suggesting that it performs comparable functions [2]. At later stages of pectoral fin outgrowth, *shh* is activated in a posterior domain of the fin bud, defining a ZPA-like region in zebrafish that appears to control AP patterning, in a manner similar to that described for chick and mouse [3], [4], [5]. Tetrapod DV patterning relies on the activity of *Wnt7a* in the dorsal ectoderm to specify dorsal structures [6], [7] and of *En1* in the ventral ectoderm to allow ventral fates to be generated in the limb bud [8]. The fact that the homologous genes are expressed in similar territories within the zebrafish pectoral fin buds, suggests a conserved role in DV patterning [9], [10].

Although important, Fgf, Hh and Wnt signalling are insufficient to account for the diversity of appendages patterning between species. Numerous studies in tetrapods provide gene expression and functional data compatible with a role for Notch signalling in several steps of limb development.

Notch is a transmembrane receptor that, upon binding to its ligands (Delta or Jagged), suffers a series of proteolytic events that result in the cleavage of the Notch intracellular domain (NICD) by gamma-secretase. NICD is then translocated to the nucleus where it associates with the DNA-binding transcriptional repressor CSL (RBPjk, Supressor of Hairless, Lag-1) turning it into a transcriptional activator, which then drives transcription of Notch target genes, such as bHLH transcription factors of the Hairy/Enhancer of Split (Hes) homologues or Hes-related (Her) family in vertebrates. Importantly, activation of the Notch receptor by its ligands requires the addition of ubiquitin to the ligand and this ubiquitination is performed by E3 ubiquitin ligase Mindbomb [11], [12].

In the tetrapod limb, it was shown that the expression of one Notch downstream target gene is dynamic and oscillates with a periodicity of 6 hours in the distal forelimb mesenchyme, suggesting that a Notch-dependent molecular clock governs the timing of formation of autopod skeletal elements [13]. In addition, Notch signalling seems to be required for a variety of functions including AER signalling [14], [15], [16], chondrogenic differentiation [17], [18] and myogenesis, a process that leads to skeletal muscle formation [19], [20].

A resident progenitor population expressing Pax3 and/or Pax7 is maintained in the developing skeletal muscle [21], [22]. Later in development the progenitor population generates satellite cells, which are marked by the expression of *Pax7* [21], [22]. Therefore, in developing and adult muscle, pools of undifferentiated cells are preserved in a latent state to undergo myogenic differentiation that allows growth and/or regeneration of muscle fibres. Importantly, controlled myogenic differentiation and maintenance of progenitors in skeletal muscles has been shown to require Notch signalling [20], [23], [24]. Nevertheless, the function of the Notch pathway during early limb and pectoral fin development is still unknown.

Here we show that several components of the Notch pathway are expressed in the myogenic mesenchyme of zebrafish pectoral fins. Without Notch signalling the muscle fibres become thin, wavy and fragmented and no stress fibres are formed in endoskeletal disc cells. Desmin and Vinculin proteins lose their normal localization in pectoral fins, indicating that the muscle fibres formed in the absence of Notch signalling produce weak mechanical forces. We also observed a decrease in the number of Pax7-positive myogenic progenitor cells in Notch signalling disrupted pectoral fins. We propose that the lack of integrity of the muscle fibres observed might be due to altered myogenesis that results in a premature reduction of myogenic progenitor cells.

## Results

### Core Elements of the Notch Pathway are Expressed in Pectoral Fin Myogenic Mesenchyme

In zebrafish, the pectoral fins arise at 24 hours-post-fertilization (hpf) as small buds of mesenchymal cells on each side of the trunk. At this time point, little or no expression of the Notch ligand *jagged2*, the Notch transmembrane receptors *notch1a*, *notch2* and *notch3* and the direct Notch targets *her6*, *her7* and *her13.2* was detected by whole mount in situ hybridization at the level of the pectoral fin buds. Later in development, at 36 hpf, when the main signalling centres of the pectoral fin are established, expression of *jagged2*, *notch1a*, *notch2, notch3*, *her6*, *her7* and *her13.2* was detected broadly in the pectoral fin (Fig. S1A–G).

**Figure 1:**
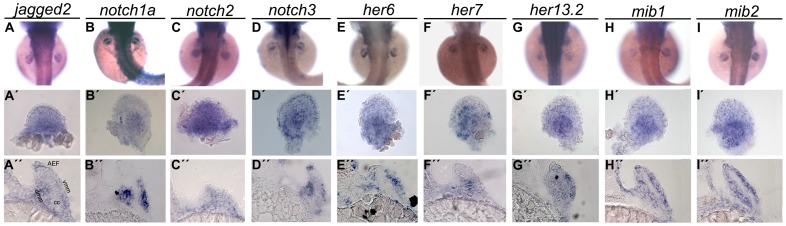
Expression pattern of Notch signalling pathway genes during pectoral fin development. Whole-mount in situ hybridization of 48 hpf embryos shows strong expression of *jagged2* (n = 20) (A), *notch1a* (n = 22) (B), *notch2* (n = 22) (C), *notch3* (n = 20) (D), *her6* (n = 25) (E), *her7* (n = 23) (F), *her13.2* (n = 23) (G), *mib1* (n = 22) (H) and *mib2* (n = 25) (I) in the pectoral fins. At this time point it is possible to detect expression of *jagged2* (A’, A”) and *notch3* (D’, D”) lining the base of the apical ectodermal fold and in the myogenic mesenchyme, *notch1a* (B’, B”), *her6* (L’, L”), *her13.2* (N’, N”), *mib1* (H’, H”) and *mib2* (I’, I”) in the myogenic mesenchyme and *notch2* (J’, J”) and *her7* (M’, M”) in the entire fin. (A’–I’) Detached pectoral fins with distal to the top. (A”-I”) Transversal sections at the level of pectoral fins. AEF, apical ectodermal fold; dmm, dorsal myogenic mesenchyme; vmm, ventral myogenic mesenchyme; cc, chondrogenic condensation.

At 48 hpf, the centre of the fin bud is occupied by a chondrogenic condensation, that will form the endoskeletal disc. This condensation divides the mesenchymal cell population into a dorsal and a ventral myogenic mesenchyme that will give rise to the fin musculature [25]. Histological sections at this time point showed expression of *jagged2*, *notch1a, notch3*, *her6* and *her13.2* in the myogenic mesenchyme (Fig. 1A”, B”, D”, E”, G”). In addition, *jagged2* and *notch3* were also expressed in the base of the apical ectodermal fold (AEF) (Fig. 1A’, A”, D’, D”). Expression of *notch2* and *her7* seems to be present in the entire fin (Fig. 1C”, F”).

An additional important component of the Notch signalling pathway is Mind bomb (Mib). This E3 ubiquitin ligase is essential for activation of Notch signalling as it promotes ubiquitination and internalization of Notch ligands [11], [12]. Our analysis reveals that both zebrafish *mib* genes (*mib1* and *mib2*) are also expressed in the myogenic mesenchyme in the pectoral fin (Fig. 1H”, I”).

At 72 hpf the expression patterns of all these genes were maintained, but they were no longer detected at later stages (Fig. S1H-N). The expression pattern of other Notch-related genes such as the ligands *deltaC* and *deltaD* and the direct targets *her1*, *her11*, *her12* and *her15* was also studied, but no expression was detected in zebrafish pectoral fins (data not shown).

Combining our data with the previously reported ubiquitous co-expression of the two *Supressor-of-Hairless Su(H)* paralogs *Su(H)1* and *Su(H)2* [26] that are essential components of the transcriptional activation complex downstream of Notch, we conclude that the core elements necessary for a functional Notch pathway are transiently expressed in the developing pectoral fin.

### Defective Pectoral Fins are Formed Upon Notch Signalling Perturbation

**Figure 2:**
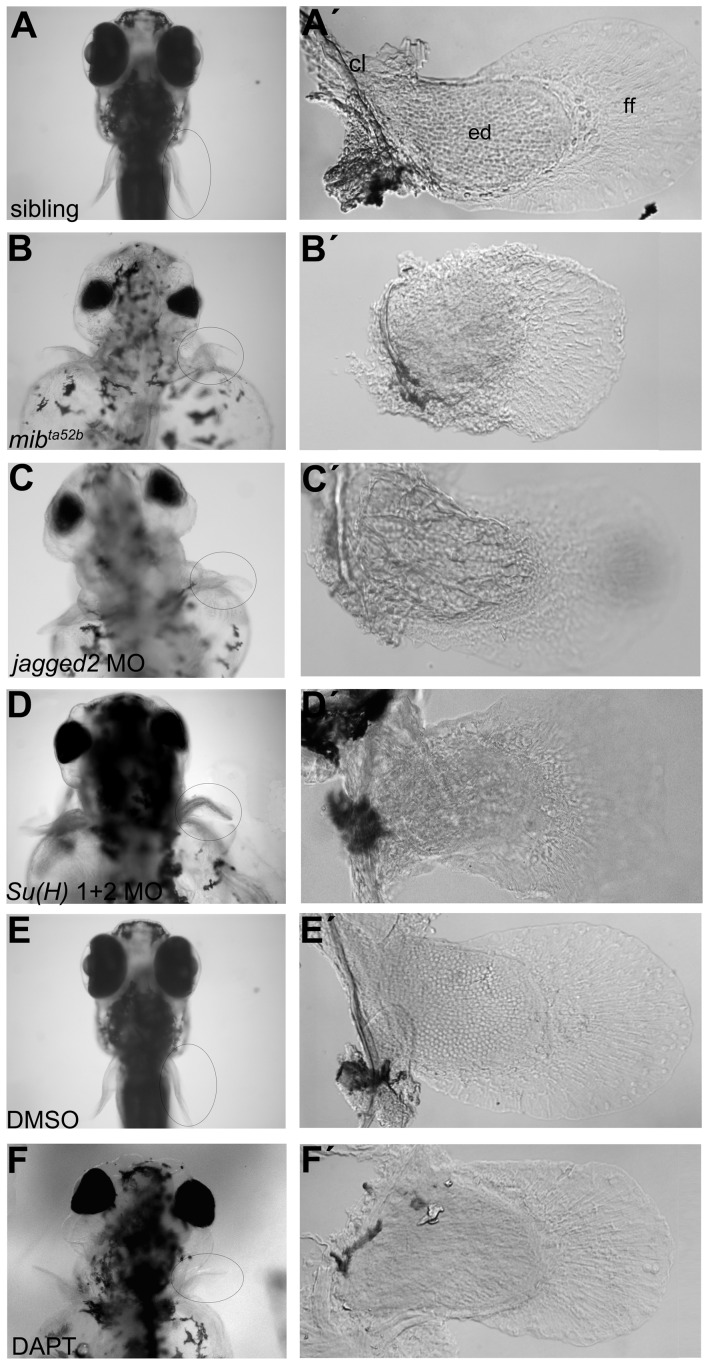
Abnormal pectoral fins are formed in Notch signalling disrupted larvae. (A–F) Live pictures of 5 dpf larvae. (A’) A pectoral fin from a sibling embryo showing the cartilaginous endoskeletal disc with individualized cells surrounded by thin matrix deposits, the fin fold and the chleitrum (n = 17) (E, E’). A similar pectoral fin was found in a DMSO-treated embryo (n = 9). Pectoral fins of Notch signalling disrupted embryos such as *mib* ^ta52b^ (n = 18) (B, B’), *jagged2* (n = 10) (C, C’) and *Su(H)1+2* (n = 12) (D, D’) morphants and DAPT-treated embryos (n = 10) (F, F’) showing disorganized endoskeletal disc cells. cl, chleitrum; ed, endoskeletal disc; ff, fin fold.

To uncover the role of Notch signalling during pectoral fin development, we started by analysing the gross pectoral fin morphology of the *mib* ^ta52b^, a severe Notch signalling mutant in which both *mib1* and *mib2* are affected [12], [27]. A sibling control pectoral fin, at 5 days-post-fertilization (dpf), is composed of a cartilaginous endoskeletal disc with individual cells surrounded by thin matrix deposits and a large fin fold supported by dermal fin rays with a characteristic open shape (Fig. 2A, A’) [25]. In clear contrast, 5 dpf *mib* ^ta52b^ mutant larvae show pectoral fins with a clear disorganized endoskeletal disc and a misshapen fin fold (Fig. 2B, B’). In addition, we used previously validated antisense morpholinos to block the Notch pathway at several levels, namely the *jagged2* ligand [28] and the two *Su(H)* genes downstream of Notch receptor [26]. In both *jagged2* and *Su(H)1+2* morphants, pectoral fins with disorganized endoskeletal disc cells were observed in 5 dpf larvae, similar to the *mib* ^ta52b^ mutant phenotype (compare Fig. 2C–D’ with Fig. 2B, B’). Thus, correct Notch pathway activity is essential for normal pectoral fin formation.

Early interference with Notch signalling, which controls several early developmental processes [29], might have indirect effects that later impact on pectoral fin formation. To examine the importance of Notch signalling at developmental stages closer to the time of fin formation, we made use of a known γ-secretase inhibitor drug called DAPT to fully block Notch signalling in a time-controlled manner [30]. We compared the pectoral fin phenotype at 5 dpf obtained when DAPT was added to the embryo medium at the 1-cell stage with that obtained when DAPT was added at 21 hpf, when pectoral fin formation is initiated. Control DMSO-treated embryos developed normal fins (Fig. 2E, E’), whereas all DAPT-treated embryos treated at the 1-cell stage or at 21 hpf showed a endoskeletal disc cells disorganization similar to that in *mib* ^ta52b^ mutants, and *jagged2* and *Su(H)1+2* morphants (Fig. 2F, F’). These experiments strongly suggest that the pectoral fin phenotype described when Notch signalling is perturbed results from an effect on fin development. Altogether, these results show that canonical Notch signalling is required for proper development of pectoral fins in zebrafish.

### Patterning and Cell Lineage Specification in the Pectoral Fins are not Affected in the Absence of Notch Signalling

To investigate whether the fin developmental defects upon Notch signalling perturbation result from an earlier disruption of the AEF and/or ZPA signalling centres, we examined the expression of key components of the Fgf and Hh pathways. In *mib* ^ta52b^ mutants the expression of *fgf8a* and *fgf24* is restricted to AEF, as in siblings (Fig. S2A–D’). *mkp3*, a readout of Fgf activity, is expressed in a PD gradient, as expected, but is slightly up-regulated proximally in the *mib* ^ta52b^ mutants (Fig. S2E-F). The expression pattern of *shh*, *ptc1* and *gli1* are unaffected in the *mib* ^ta52b^ mutants. In both *mib* ^ta52b^ mutants and their siblings, the expression of *shh* and its receptor *ptc1* appears restricted to the ZPA (Fig. S2G-J’) and *gli1,* a readout of Hh activity, is expressed in the fin mesenchyme (Fig. S2K-L’). As in tetrapods, signals from the AEF and the ZPA might be able to regulate *hox* gene expression in zebrafish and in this way specify regional identity along the fin [31]. Once again, no alteration in the expression of *hoxa9b*, *hoxa11b* and *hoxa13a* was observed between the *mib* ^ta52b^ mutants and their siblings (Fig. S2M-R’). This analysis demonstrates that patterning along the PD and AP axes of the pectoral fin is broadly unaffected upon Notch signalling impairment.

Our data show that several Notch signalling components were expressed at early stages of pectoral fin development at the level of the myogenic mesenchyme (Fig. 1), raising the possibility that this pathway might be important to define muscle versus cartilage lineages within the pectoral fin.

**Figure 3:**
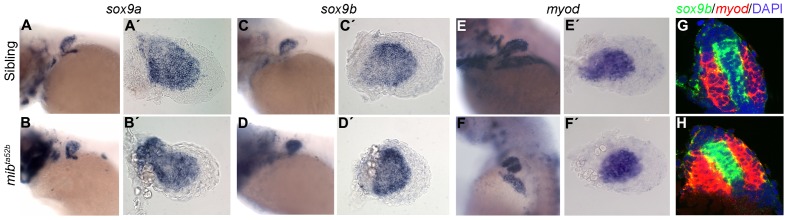
Cartilage and muscle masses are specified in *mib* ^ta52b^ mutants. Whole-mount in situ hybridization of 68 hpf siblings (n = 15) (A, A’), (n = 12) (C, C’), (n = 14) (E, E’) and *mib^ta52b^* mutants (n = 12) (B, B’), (n = 15) (D, D’), (n = 11) (F, F’) using *sox9a* and *sox9b* as cartilage markers and *myod* as a muscle marker, showing a slight upregulation of these genes in *mib^ta52b^* mutant pectoral fin. (A–F) Whole larvae. (A’–F’) Detached pectoral fins with distal to the right. Double fluorescent in situ hybridization on sections using *sox9b* (green) and *myod* (red), counterstained with DAPI to label the nuclei (blue) shows no signs of cell mixing in a sibling (n = 7) (G) and a *mib^ta52b^* mutant pectoral fins (n = 9) (H). An upregulation of these genes is observed in *mib^ta52b^* mutant pectoral fins when compared with the sibling.

To address the possibility that Notch signalling interferes with early stages of muscle or cartilage differentiation, we performed an in situ hybridization analysis using *myod*, an early marker of myogenic differentiation [32], and *sox9a* and *sox9b*, cartilage markers [33]. At 68 hpf, *sox9a*, *sox9b* and *myod* positive cells were detected within the fins of *mib* ^ta52b^ mutants and their siblings (Fig. 3A–F’), indicating that both pectoral fin cell lineages are specified in the absence of Notch signalling. Moreover, in a double fluorescent in situ analysis with *sox9b* and *myod*, we found no signs of cartilage and muscle lineages cell mixing in the absence of Notch signalling (Fig. 3G, H). Overall, these results suggest that defects of patterning, cell lineage specification or muscle precursor ingression are not the cause of the pectoral fin phenotype observed when Notch signalling is perturbed.

### Notch Signalling Impacts on Skeletal Muscle Fibre Integrity and Stress Fibre Formation in Pectoral Fins

To characterize with cellular resolution the pectoral fin architecture later in development, after tissue differentiation has occurred, we used DAPI and phalloidin to label cell nuclei and filamentous actin, respectively.

**Figure 4:**
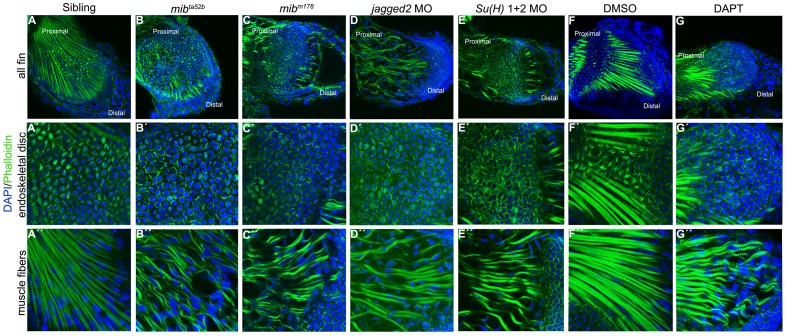
Notch signalling is crucial for skeletal muscle integrity and stress fibres formation. Immunostaining of 5 dpf pectoral fins (A–G”) with DAPI and phalloidin to label nuclei (blue) and filamentous actin (green), respectively. Stress fibres in the endoskeletal disc cells and intact skeletal muscle fibres are formed in the pectoral fins of siblings (n = 20) (A–A”) and in DMSO-treated embryos (n = 17) (F-F”). In the pectoral fins were Notch signalling was disrupted like in *mib* ^ta52b^ (n = 22) (B–B”) and *mib* ^m178^ (n = 10) (C–C”) mutants, *jagged2* (n = 11) (D–D”) and *Su(H)*1+2 (n = 10) (E–E”) morphants and in DAPT-treated embryos (n = 16) (G–G”) the endoskeletal disc cells present high levels of actin at the periphery and the skeletal muscle fibres are wavy with gaps between them.

In control embryos at 3 (data not shown) and 5 dpf, the endoskeletal disc cells possess an actin cytoskeleton organized in transversal stress fibres (Fig. 4A’, F’). In *mib* ^ta52b^ and *mib* ^m178^ mutants, in *jagged2* and *Su(H)1+2* morphants and in DAPT-treated embryos, the actin organization was distinct from controls; the actin filaments accumulated at the periphery of the endoskeletal disc cells (Fig. 4B’–E’, G’).

The endoskeletal disc separates the fin musculature into two opposing muscles, the abductor and the adductor. Within these muscles, the individual muscle fibres run in sheets in a semiradial fashion along the PD axis of the muscles [34]. This was exactly what we found in both 3 dpf (data not shown) and 5 dpf control pectoral fins, where well-aligned striated muscle fibres extend along the PD axis (Fig. 4A”, F”). In *mib* ^ta52b^ and *mib* ^m178^ mutants, in *jagged2* and *Su(H)1+2* morphants and in DAPT-treated embryos, striated muscle fibres are formed. However, the fibres are thinner, wavy and fragmented, leading to gaps in the fin musculature (Fig. 4B”–E”, G”).

**Figure 5:**
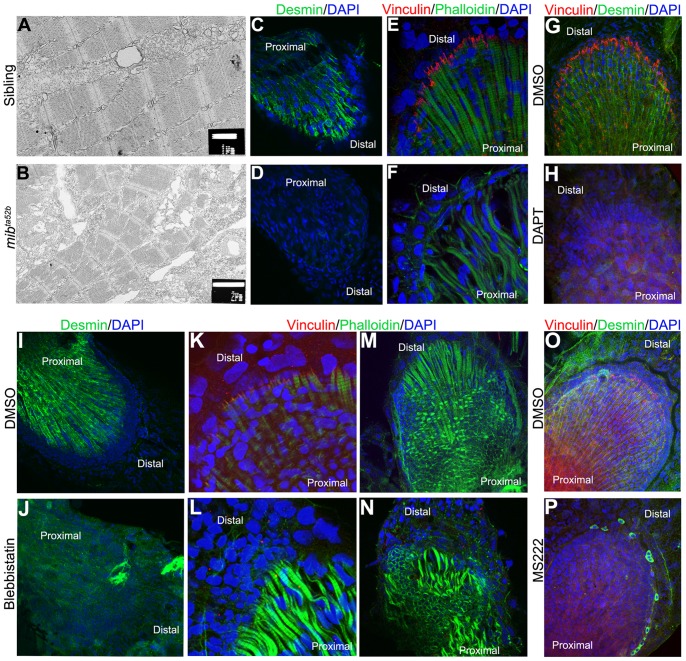
Mechanical weak muscles fibres are produced when Notch signalling is perturbed. Transmission electronic microscopy was performed in 5 dpf pectoral fins of siblings (A) and *mib* ^ta52b^ mutants (B) to analyse the ultrastructure of skeletal muscle fibres. The myofibrils with clear aligned sarcomeres are formed in the sibling embryo (A), while disintegrating myofibrils with poorly aligned sarcomeres are found in *mib* ^ta52b^ mutants (B). Immunostaining performed in pectoral fins at 5 dpf using Desmin, Vinculin, phalloidin and DAPI to label the Z-discs of the sarcomeres, the zone where the skeletal muscle fibres insert distally, filamentous actin and the cell nuclei, respectively (C–P) demonstrate that Desmin (n = 10) (D) and Vinculin (n = 12) (F) are downregulated in *mib* ^ta52b^ mutants when compared with their siblings (n = 10) (C, E). The same is observed in DAPT-treated embryos at 48 hpf and fixed at 5 dpf (n = 9) (H), when compared with the DMSO-treated embryos (n = 10) (G). In embryos treated with blebbistatin, Desmin (n = 9) and Vinculin (n = 12) are also downregulated (J, L) when compared with the DMSO-treated embryos (n = 12) (I, K). In blebbistatin-treated embryos, the endoskeletal disc cells present high levels of actin at the periphery and the skeletal muscle fibres are wavy with gaps between them (n = 15) (N). A down-regulation of Desmin and Vinculin is also observed in MS222-treated embryos (n = 14) (P) when compared with the control embryos (n = 13) (O).

We used transmission electron microscopy to characterize the skeletal muscle fibre phenotype observed in the *mib* ^ta52b^ mutants. We found that, in contrast to the sibling pectoral fins, which show well-organized myofibrils with sarcomeres in register, *mib* ^ta52b^ mutants show severely disrupted myofibrils. Although these myofibrils have sarcomeric organization, they are fragmentary and are often separated by large areas of cytoplasm (compare Fig. 5A with Fig. 5B). These results demonstrate that Notch signalling is essential for both muscle integrity and skeletal architecture in pectoral fins.

### Mechanically Fragile Skeletal Muscle Fibres are Formed in Pectoral Fins in the Absence of Notch Signalling

The lack of integrity of the myofibrils that we uncovered in *mib* ^ta52b^ mutants could be due to a mis-regulation of the muscle specific intermediate filament protein Desmin. It has been shown that Desmin localizes to the Z-discs of the sarcomeres and plays a fundamental role in maintaining the integrity of the myofibrils and their connection to the subsarcolemmal cytoskeleton, thereby ensuring mechanically resilient muscles [35], [36].

**Figure 6:**
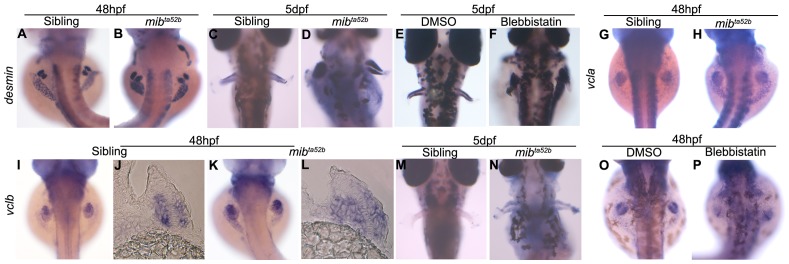
*desmin* and *vinculin* are not transcriptionally downregulated in *mib* ^ta52b^ mutants. Whole-mount in situ hybridization using *desmin*, *vcla* and *vclb* as mRNA probes. At 48 hpf, *desmin* is expressed at similar levels in the myogenic mesenchyme of the pectoral fins of siblings (n = 18) (A) and *mib* ^ta52b^ mutants (n = 20) (B). At 5 dpf, the expression of *desmin* becomes stronger in *mib* ^ta52b^ mutants (n = 19) (D) when compared with the sibling (n = 16) (C). Blebbistatin-treated embryos (n = 12) (F) show a similar level of *desmin* expression as control DMSO-treated (n = 10) (E). *vcla* is expressed strongly in the somites and has a faint expression in the sibling pectoral fins (n = 15) (G), the same expression pattern can be observed in the *mib* ^ta52b^ mutant (n = 18) (H). *vclb* is expressed in the myogenic mesenchyme at 48 hpf in both siblings (n = 18) (I), (n = 5) (J) and *mib* ^ta52b^ mutants (n = 19) (K), (n = 5) (L). At 5 dpf, the expression of *vclb* is poorly detected in the sibling embryos (n = 15) (M) and is still detected in *mib* ^ta52b^ mutants (n = 16) (N). Transversal sections at the level of the pectoral fin of 48 hpf embryos are shown (J, L). Blebbistatin-treated embryos (n = 10) (P) show a similar level of *vclb* expression as control DMSO-treated (n = 12) (O).

By using immunohistochemistry, we were able to show that Desmin protein is abundantly expressed in the skeletal muscle fibres of 5 dpf sibling and DMSO-treated pectoral fins (Fig. 5C, G), but is severely downregulated in *mib* ^ta52b^ mutants and DAPT-treated embryos (Fig. 5D, H). Interestingly, lack of Desmin protein is not a consequence of lack of *desmin* mRNA transcription as detected by in situ hybridization in *mib* ^ta52b^ mutants pectoral fins (Fig. 6B, D). These results suggest that *desmin* might not be the primary cause of the muscle phenotype in *mib* ^ta52b^ mutants but unveil the possibility, amongst others, that forces generated by mechanically stable muscle fibres are essential to maintain appropriate Desmin protein levels.

Myotendinous junctions (MTJ) form a mechanical unit that provides structural stability linking muscle fibres and extracellular matrix molecules (ECM) [37]. Therefore, structurally unstable muscle fibres could result from impaired formation/function of the MTJ.

In the zebrafish pectoral fin, the two opposing muscles originate from a proximal bone called the cleithrum and insert distally in the fin membrane at the end of the endoskeletal disc [34], presumably through a MTJ-type linkage. As the MTJ has not been characterized in zebrafish fins, we looked for components of this type of linkage system in 5 dpf control pectoral fins. We found that Vinculin, a focal adhesion protein involved in linking actin filaments in cytoplasm through integrins to the ECM molecules in the extracellular space [38], is localized in the pectoral fin where muscle fibres are thought to insert distally in wild type control embryos (Fig. 5E). In contrast, Vinculin is not detected at the distal end of the muscle fibres in *mib* ^ta52b^ mutants and DAPT-treated embryos (Fig. 5F, H). Thus, abrogation of Notch signalling leads to defects in MTJ assembly.

Vinculin recruitment to focal adhesions is force-dependent [38], [39] raising the possibility that loss of Vinculin in the pectoral fins could be a consequence of the muscle phenotype and not its primary cause. To distinguish between these two possibilities we started by looking at the mRNA expression of *vinculin* (*vcl*) in pectoral fins of control and *mib* ^ta52b^ mutant embryos. In zebrafish, there are two *vcl* genes (Leslie, Hinits, Williams and Hughes; unpublished data). *vcla* is expressed in the somites and in pectoral fins (Fig. 6G) and *vclb* in the myogenic mesenchyme of pectoral fins (Fig. 6I, J, M). Expression of both *vcla* and *vclb* mRNAs occurs in *mib* ^ta52b^ mutants (Fig. 6H, K, L, N), showing that Notch signalling does not act via transcriptional control of Vinculin genes.

We tested independently the possibility that impaired mechanical forces produced by unstable muscle fibres impact on the stabilization of Desmin and Vinculin protein levels. To that end, we inhibited muscle contraction and therefore force generation using the myosin-inhibitor blebbistatin [40] and MS222, a muscle relaxant that operates by preventing action potentials [41]. We added blebbistatin or MS222 to wild-type embryos at 2 dpf and performed an immunostaining to detect the localization of Desmin and Vinculin at 5 dpf. Control embryos showed the expected striated Desmin expression (Fig. 5I, O) and Vinculin localization at the distal end of the pectoral fin muscle fibres (Fig. 5K, O), whereas in the blebbistatin and MS222-treated embryos Desmin was severally reduced (Fig. 5J, P) and Vinculin accumulation was not visible (Fig. 5L, P). Similar to what we observed in the *mib* ^ta52b^ mutants, the transcription of *desmin* and *vclb* is not affected in blebbistatin-treated embryos (Fig. 6E, F, O, P).

This further supports the idea that the lack of Desmin and Vinculin in the pectoral fin seen in the absence of Notch signalling is a consequence of the muscle phenotype. Moreover, it also shows in the context of a living embryo that the accumulation of these proteins is dependent on mechanical forces produced by the skeletal muscles.

Interestingly, we could also see that in blebbistatin and MS222-treated embryos, actin filaments are accumulated at the periphery of the endoskeletal disc cells (Fig. 5N, compare with 5M and data not shown), reminiscent of the effect produce by the absence of Notch signalling (Fig. 4B’–E’, G’).

### Notch Signalling is Required to Set up a Pax7 Myogenic Progenitor Cell Population in Pectoral Fins

**Figure 7:**
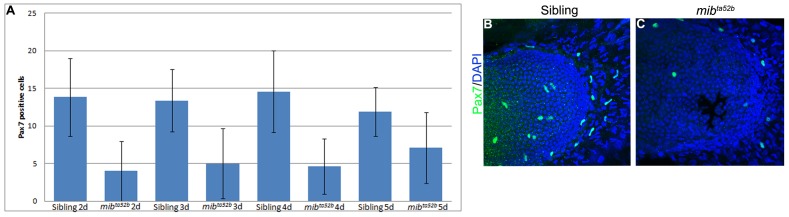
Pax7 muscle progenitor cells are depleted in *mib* ^ta52b^ mutant pectoral fins. (A) Number of Pax7-positive cells in siblings and *mib* ^ta52b^ mutants at time points 2 dpf (sibling n = 6, *mib* ^ta52b^ n = 7, t-test p = 0.004), 3 dpf (sibling n = 13, *mib* ^ta52b^ n = 10, t-test p = 0.0002), 4 dpf sibling n = 7, *mib* ^ta52b^ n = 14, t-test p = 0.0019) and 5 dpf (sibling n = 14, *mib^ta52b^* n = 15, t-test p = 0.003). Error bars = SD. 5 dpf pectoral fins immunostained for Pax7 and DAPI to label muscle progenitor cells and nuclei, respectively, in (B) siblings and (C) *mib* ^ta52b^ mutants.

The lack of integrity of the muscle fibres observed in the zebrafish *mib* ^ta52b^ mutants could result from an inefficient muscle differentiation due to premature uncontrolled differentiation accompanied by depletion of progenitor cells. To test this possibility, we performed immunohistochemistry with the paired-box transcription factor Pax7, a marker of proliferating muscle progenitor cells. We observed a severe reduction in the number of Pax7-positive cells in the *mib* ^ta52b^ mutant pectoral fins when compared with siblings in all time points analysed. At 2 dpf, an average of 4 Pax7-positive cells were found in *mib* ^ta52b^ mutants (n = 7) in contrast to siblings that showed an average of 13.8 (n = 6). At 3 dpf, we counted on average 5 Pax7-positive cells in *mib* ^ta52b^ mutants (n = 10) and on average of 13.3 Pax7-positive cells in their siblings (n = 13). At 4 dpf an average of 4.6 Pax7-positive cells were counted in *mib* ^ta52b^ mutants (n = 14) and an average of 14.5 Pax7-positive cells was founded in their siblings (n = 7). At 5 dpf, the *mib* ^ta52b^ mutants showed on average 7 (n = 15) Pax7-positive cells, significantly fewer than the 11.9 (n = 14) in siblings at this stage (Fig. 7A–C). These results suggest that Notch signalling is required early on to establish a Pax7 myogenic progenitor cell pool.

## Discussion

Fish pectoral fins are, in many respects, homologues of tetrapod forelimbs. However, a clear contrast exists concerning the role of Notch signalling during pectoral fin development.

We started by performing a thorough expression analysis of the Notch pathway components in developing pectoral fin buds. We show that the core components needed for a functional Notch pathway are expressed in pectoral fin buds at similar stages. Namely, we found that the Notch ligand *jagged2*, the transmembrane receptors *notch1a*, *notch2* and *notch3*, the Notch direct targets *her6*, *her7* and *her13.2* and the E3 ubiquitin ligases *mib1* and *mib2* are all expressed in the entire fin bud at 36 hpf. Whereas mRNA of *notch2* and *her7* is present in the entire fin bud at 48 hpf, we found that expression of *jagged2*, *notch1a*, *notch3*, *her6*, *her13.2*, *mib1* and *mib2* becomes restricted to the myogenic mesenchyme. We also found expression of *jagged2* and *notch3* at the apical ectodermal fold (AEF) at this later stage. The expression pattern of all these genes is maintained at least until 72 hpf. However, and in contrast to the situation in the chick embryo where cyclic gene expression was described for the Notch-based clock gene *hairy2* [13], we were unable to detect any sign of dynamic gene expression of any of the zebrafish cyclic genes, namely *her1*, *her7*, *her12* and *her15* in the developing pectoral fin. Nevertheless, the expression of several elements of the Notch pathway in the myogenic region and in the apical ectodermal ridge (AER) in fish pectoral fins was similar to that reported in developing chick and mouse limbs [15], [16], [19], [20], suggesting a conserved role for Notch signalling in vertebrate appendage development.

We used a strong Notch signalling mutant allele (*mib^ta52b^*) [12], [27] to dissect the function of the Notch pathway in pectoral fin development. We started by analysing the impact that Notch signalling could have in the early establishment of signalling centres, namely the AEF and the ZPA, as these are crucial to pattern the fin along the PD and the AP axes, respectively. We found that in the absence of Notch signalling the majority of the components of Fgf signalling pathway that are mediators of AEF function and the Hh pathway that are mediators of the ZPA function appear normal. Fitting with these data is the fact that a normal AEF and ZPA function observed in the absence of Notch signalling translates into a normal regional identity along the fin bud, as evaluated by the expression of *hox* genes.

In the absence of Notch signalling we also found that the early specification of cartilage and muscle lineages was apparently normal in the pectoral fins, as we could detect expression of *sox9a*/*b* and *myod* in the correct territories, respectively. This also compares with the results described for *notch1*, *jagged2* and *delta1* mouse mutants, in which no muscle or cartilage/bone specification problems were reported [14], [15], [16], [20]. Thus, we did not detect an early patterning/specification phenotype in the pectoral fins of *mib^ta52b^* mutants.

A striking phenotype was observed in Notch signalling-defective muscle at 3–5 dpf. Gaps in the fin musculature were detected in *mib^ta52b^* mutants that seem to result from the formation of thin, wavy and fragmented striated muscle fibres. At the ultrastructural level, we found myofibrils with recognisable sarcomeres present in *mib^ta52b^* mutant muscle fibres. However, their integrity was severely disrupted.

A role for Mib2 in maintaining the integrity of fully differentiated muscles was shown in *Drosophila*. In the absence of *mib2*, apoptotic degeneration [42] and sarcomere instability [43] occur in skeletal muscles. Interestingly, Mib2 seems to maintain muscle integrity in a novel Notch-independent pathway, as *mib1* fails to rescue the muscle mutant phenotype of *mib2* in *Drosophila* [42], [43]. These results in *Drosophila* raised the possibility that the skeletal muscle phenotype that we have uncovered in the *mib^ta52b^* mutant allele could be due to Mib2 in a Notch-independent manner, since both *mib1* and *mib2* are affected in this mutant allele [12], [27]. Therefore, we analysed the mutant allele *mib^m178^*, where only *mib1* is affected [11], [44], [45], and showed that thin, wavy and fragmented striated muscle fibres are formed. In addition, we downregulated the ligand *jagged2* and the transcriptional components *Su(H)1+2* downstream of the Notch receptor and used the DAPT drug to block Notch signalling in a time-controlled manner. In all these situations a similar severe disruption of the skeletal muscle fibres was observed in the pectoral fins at 3–5 dpf. Therefore, we conclude that in zebrafish canonical Notch signalling through the *jagged2* ligand is essential to promote the integrity of the muscle fibres.

To dissect further the reason behind the muscle phenotype in *mib^ta52b^* mutants, we examined expression of the muscle cytoskeletal components, Desmin and Vinculin. Desmin is an intermediate filament protein that anchors the myofibrils to each other and to the subsarcolemmal cytoskeleton [35], [36] and Vinculin is a focal adhesion molecule involved in the anchorage of actin filaments of the muscle fibres to the extracellular matrix [38]. Although the genes encoding Desmin and Vinculin were apparently transcribed normally, we detected a severe downregulation of Desmin and Vinculin protein levels in *mib^ta52b^* mutant pectoral fins. As recruitment of Vinculin to focal adhesions is partly force-dependent, our results raise the possibility that in the absence of Notch signalling the muscles fibres do not generate sufficient mechanical force to localize/stabilize Vinculin to the distal end of the muscle fibres. The similar behaviour of Desmin suggests that the accumulation of this protein could also be dependent on forces generated by the muscle fibres. In fact, we show in an independent manner that, in embryos where muscle contraction was inhibited using blebbistatin or MS222, the levels of Desmin and Vinculin proteins were severely downregulated, even though the levels of mRNA were not affected.

Why might Notch signalling defects lead to weak muscle fibres and subsequent failure of cytoskeletal maturation? We found a decrease of Pax7-positive cells in *mib^ta52b^* mutants from early stages of pectoral fin development, suggestive of a premature uncontrolled differentiation of myogenic progenitors, as has been shown in other contexts in which Notch signalling was blocked [20], [23], [24].

We propose that altered myogenesis in the absence of Notch signalling in *mib^ta52b^* mutant pectoral fins, reflected by the lack of Pax7-positive cells, might underly defective muscle fibre generation, which in turn triggers a failure of normal muscle cytoskeletal maturation.

An additional indication that the muscle fibres formed in pectoral fins in the absence of Notch signalling are unable to generate normal mechanical forces is the observation that actin stress fibres are not formed in endoskeletal disc cells. A similar actin defect is observed in immotile blebbistatin and MS222-treated embryos. It has been shown that external forces applied to the cytoskeleton cause the formation of stress fibres, as force promotes actin filament aggregation in an orientation parallel to the direction of the force application [46]. Interestingly, several studies have found evidence for a relationship between muscle force and bone formation/integrity [32], [47], [48], [49], [50], [51], [52], [53]. However, without Notch signalling our embryos do not survive beyond 5 dpf, so we were unable to determine the effect of lack of stress fibres in the endoskeleton on the later formation of bone elements in pectoral fins.

## Materials and Methods

### Ethics Statement

All experiments involving animals were approved by the Animal User and Ethical Committees at Instituto de Medicina Molecular, according with directives from Direcção Geral Veterinária (PORT1005/92) or under UK Home Office licence.

### Zebrafish Embryos

Embryos from AB lines and *mib* ^ta52b^ and *mib* ^m178^ mutants were kept at 28°C and classified accordingly to [54].

### In situ Hybridization

Single whole-mount in situs were performed as described [55]. Double whole-mount fluorescent in situs were performed as described [56] with modifications; the red signal was developed with FAST RED (Roche AP substrate) and the green with Tyramide FITC (POD substrate).

### Microinjections

Morpholinos to *Su(H)1+2* (22 ng per embryo of 5′-CAAACTTCCCTGTCACAACAGG-3′, described in [26]) and *jagged2* (12 ng per embryo of 5′-TCCTGATACAATTCCACATGCCGCC-3′, described in [28]) were purchased from Gene Tools and injected at the one-cell stage.

### Drug Treatment

DAPT (100 µM final concentration, D5942-Sigma) or DMSO control was added to embryo medium at indicated time points (1 cell-stage or 21 or 48 hpf) and then washed at 4–5 dpf. Blebbistatin (6 µM final concentration, B0560-Sigma) or DMSO control was added to embryo medium at 48 or 72 hpf and then washed at 5 dpf. MS222 (0.016%, A5040-Sigma) was added at 48 hpf to embryo medium and washed at 4–5 dpf, whereas control embryos were grown in embryo medium until 5 dpf.

### Immunohistochemistry

Whole-mount immunostainings of 3 and 5 dpf pectoral fins were performed as described [57], using Desmin (1∶80; Sigma), Vinculin (1∶500; Sigma), Pax7 (1∶5; [58]), Alexa Fluor 488 and 594 (1∶500; Invitrogen). Filamentous actin and nuclei were detected with Alexa Fluor 488 Phalloidin (1∶100; Molecular Probes) and DAPI (Sigma), respectively. At the time point of 48 hpf sibling and *mib^ta52b^* embryos were fixed in 4% PFA, embedded in 30% sucrose/PBS and then placed in 7.5% gelatin/15% sucrose. The embedded embryos were serially cryosectioned (transverse sections) at the level of pectoral fins, and immunostained on sections by washing in PBS, permeabilizing in 0.5% Trinton X-100/PBS for 20 minutes and then incubating in blocking buffer (1% bovine serum albumin in PBS) for a further 20 minutes before staining with anti-Pax7 (1∶5; [58]), Alexa Fluor 488 (1∶500; Invitrogen) and DAPI (Sigma).

### Imaging

Following the in situs, whole embryos were photographed with a LEICA Z6 APO stereoscope coupled to a LEICA DFC490 camera and detached pectoral fins and sections were photographed with a Leica DMR microscope coupled to a Leica DC500 camera. Following the immunostainings detached pectoral fins and histological sections of pectoral fins at 48 hpf were examined with a Zeiss LSM 510 Meta confocal microscope. Two and three-colour confocal z-series images were acquired using sequential laser excitation, converted into a single plane projection and analyzed using ImageJ software (LSM Reader).

### Transmission Electronic Microscopy (TEM)

For TEM analysis, wild-type and *mib* ^ta52b^ mutant at 5 dpf were fixed with 2.5% glutaraldehyde in 0.1 M sodium cacodylate buffer, post-fixed in 1% osmium tetroxide, dehydrated through an ethanol series and then embedded in Spurr’s resin. The embedded embryos were serially sectioned (transverse sections) at the level of pectoral fins using an ultramicrotome. The ultrathin sections (70 nm) were then stained with uranyl acetate and plumbum citrate and viewed using a Jeol Jem-1010 electron microscope. Figures were assembled using Adobe Photoshop CS2.

### Statistical Methods

In detached pectoral fins and histological sections of sibling and *mib^ta52b^* mutants, cells were counted manually in Pax7 and DAPI-stained preparations photographed by a Zeiss LSM 510 confocal microscope. In the case of the histological sections, the number of Pax7-positive cells was counted in each slice of sectioned pectoral fin. A two-tailed Student’s t-test was used. The statistical significance was determined as a p-value of 0.05 or less.

## Supporting Information

Figure S1 Expression pattern of Notch signalling pathway genes at early and late time points of pectoral fin development. Expression of *jagged2* (n = 25) (A), *notch1a* (n = 27) (B), *notch2* (n = 20) (C), *notch3* (n = 25) (D), *her6* (n = 22) (E), *her7* (n = 20) (F) and *her13.2* (n = 20) (G) can be detected in the entire pectoral fin at 36 hpf. Later in development at 72 hpf the expression of these genes is still detected in the detached pectoral fins with distal to the top (H–N).
(TIF)

Figure S2 Proximal-distal and anterior-posterior patterning and Hox identity are not affected in *mib* ^ta52b^ mutants. The Fgf signalling components *fgf8* (n = 15) (A, A’) and *fgf24* (n = 12) (C, C’) are expressed in the apical ectodermal fold of 48 hpf sibling embryos. The same pattern of expression is observed in the pectoral fins of *mib* ^ta52b^ mutants (n = 12) (B, B’), (n = 14) (D, D’). The Fgf signalling downstream target *mkp3* is expressed in a gradient through a distal to proximal direction in fin mesenchymal cells of sibling embryos (n = 16) (E, E’). The expression of *mkp3* is upregulated proximally in *mib* ^ta52b^ mutants (n = 18) (F, F’). The Hh signalling components *shh* and *ptc1* are expressed in the zone of polarizing activity and *gli1* in the mesenchymal cells that compose the fin. No differences in the expression pattern can be observed between siblings (n = 12) (G, G’), (n = 15) (I, I’), (n = 10) (K, K’) and *mib* ^ta52b^ mutants (n = 10) (H, H’), (n = 12) (J, J’), (n = 11) (L, L’). The expression of *hoxa9b*, *hoxa11b* and *hoxa13a* reveals no differences in the patterns of these genes in siblings (n = 9) (M, M’), (n = 11) (O, O’), (n = 10) (Q, Q’) and *mib* ^ta52b^ mutants (n = 10) (N, N’), (n = 9) (P, P’), (n = 11) (R, R’). (A–R) Whole embryos. (A’–R’) Detached pectoral fins with distal to the top and posterior to the right.
(TIF)
