# Supplementary figures and images for: Notch Signalling Is Required for the Formation of Structurally Stable Muscle Fibres in Zebrafish
Source: PLoS One. 2013 Jun 28;8(6):e68021. doi: 10.1371/journal.pone.0068021 (PMC3695967; doi:10.1371/journal.pone.0068021)

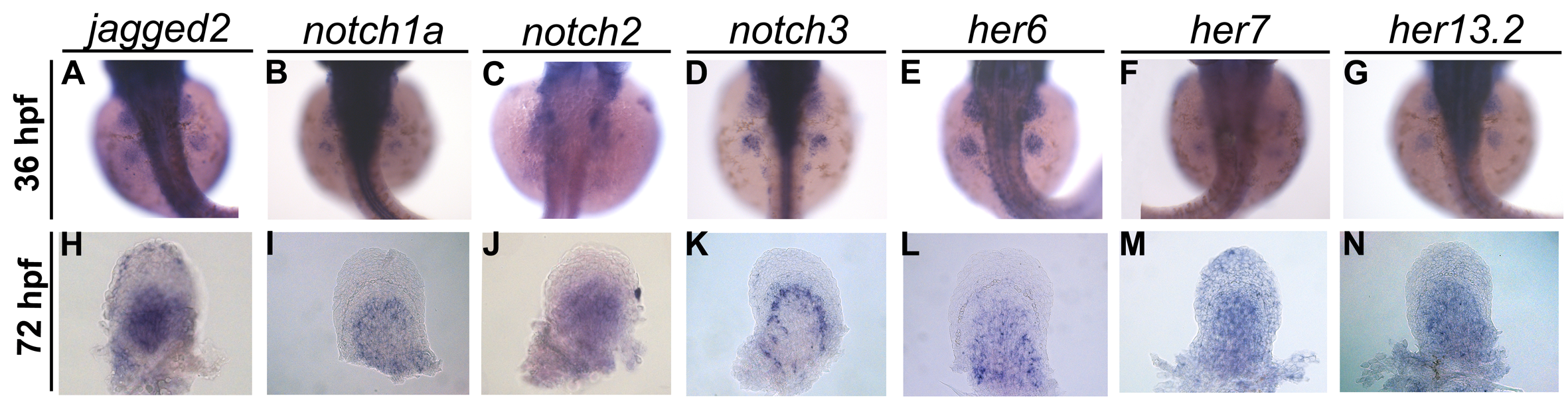

Supplement: Figure S1 — Expression pattern of Notch signalling pathway genes at early and late time points of pectoral fin development. Expression of jagged2 (n = 25) (A), notch1a (n = 27) (B), notch2 (n = 20) (C), notch3 (n = 25) (D), her6 (n = 22) (E), her7 (n = 20) (F) and her13.2 (n = 20) (G) can be detected in the entire pectoral fin at 36 hpf. Later in development at 72 hpf the expression of these genes is still detected in the detached pectoral fins with distal to the top (H–N). (TIF) [file pone.0068021.s001.tif]

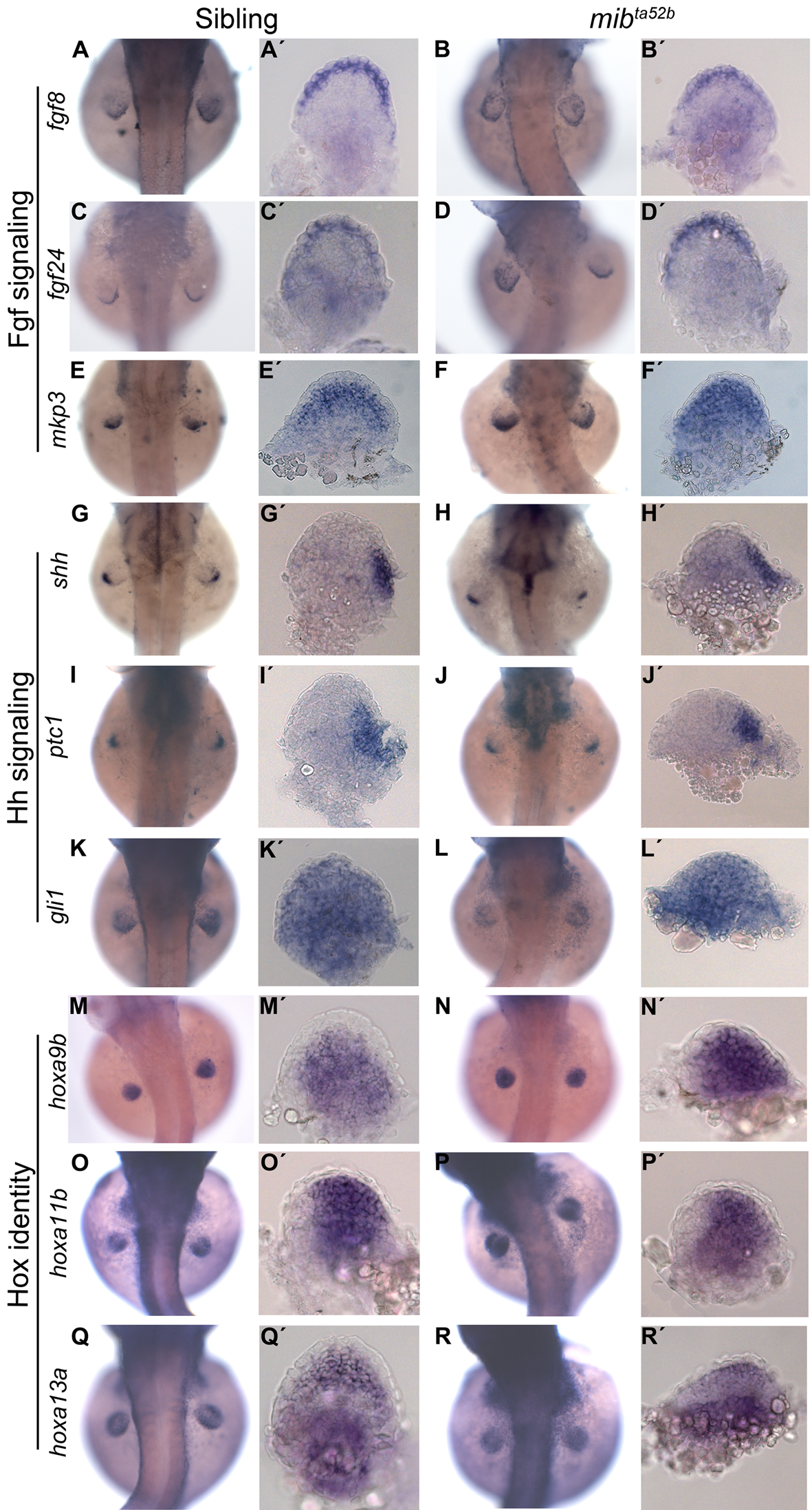

Supplement: Figure S2 — Proximal-distal and anterior-posterior patterning and Hox identity are not affected in mib ta52b mutants. The Fgf signalling components fgf8 (n = 15) (A, A’) and fgf24 (n = 12) (C, C’) are expressed in the apical ectodermal fold of 48 hpf sibling embryos. The same pattern of expression is observed in the pectoral fins of mib ta52b mutants (n = 12) (B, B’), (n = 14) (D, D’). The Fgf signalling downstream target mkp3 is expressed in a gradient through a distal to proximal direction in fin mesenchymal cells of sibling embryos (n = 16) (E, E’). The expression of mkp3 is upregulated proximally in mib ta52b mutants (n = 18) (F, F’). The Hh signalling components shh and ptc1 are expressed in the zone of polarizing activity and gli1 in the mesenchymal cells that compose the fin. No differences in the expression pattern can be observed between siblings (n = 12) (G, G’), (n = 15) (I, I’), (n = 10) (K, K’) and mib ta52b mutants (n = 10) (H, H’), (n = 12) (J, J’), (n = 11) (L, L’). The expression of hoxa9b, hoxa11b and hoxa13a reveals no differences in the patterns of these genes in siblings (n = 9) (M, M’), (n = 11) (O, O’), (n = 10) (Q, Q’) and mib ta52b mutants (n = 10) (N, N’), (n = 9) (P, P’), (n = 11) (R, R’). (A–R) Whole embryos. (A’–R’) Detached pectoral fins with distal to the top and posterior to the right. (TIF) [file pone.0068021.s002.tif]
